# [^18^F]fluorodeoxyglucose PET/MRI for nononcological musculoskeletal indications: reference uptake values in the asymptomatic lumbar spine and example comparison to a low back pain patient

**DOI:** 10.1097/MNM.0000000000002164

**Published:** 2026-04-27

**Authors:** Jacob M. Mostert, Edwin H.G. Oei, Denise W.J. van Beekveld, Sandip Biswal, Rianne A. van der Heijden

**Affiliations:** a Department of Radiology and Nuclear Medicine, Erasmus University Medical Center, Rotterdam, The Netherlands; b Department of Radiology, University of Wisconsin-Madison, Madison, Wisconsin, USA

**Keywords:** fluorodeoxyglucose, inflammation, lumbar spine, low back pain, PET/MRI

## Abstract

**Objective:**

To assess normal [^18^F]fluorodeoxyglucose ([^18^F]FDG) uptake values in lumbar spine structures on PET/MRI in patients without low back pain.

**Methods:**

Asymptomatic adults receiving whole-body FDG PET/MRI for nononcological indications were prospectively included. Spinal structures of interest were manually annotated at each lumbar level. Maximum standardized uptake value (SUV_max_) and mean SUV (SUV_mean_) values were extracted from ordered subset expectation maximization, block sequential regularized expectation maximization (BSREM) β450, and BSREM β300 images. The distributions of SUV_max_ and SUV_mean_ values were evaluated across spinal levels and structures. We also evaluated normalization of SUV metrics with the liver and subcutaneous fat uptake values, and correlations between SUV metrics and BMI, age, and blood glucose level.

**Results:**

Twenty-two patients were included for analysis. Average SUV_max_ on OSEM reconstructions was 2.99 (SD: 0.69) for the vertebral body and 1.95 (SD: 0.54) for the intervertebral disc. In the nervous system, SUV_max_ for articular, peri-articular, and ligamentous structures was lower, ranging between 1.26 and 1.62. Uptake values were significantly lower on BSREM β450 and higher on BSREM β300. There was a positive correlation between uptake values and BMI, but no correlation was found with age or blood glucose level.

**Conclusion:**

Normal uptake values in the lumbar spine on [^18^F]FDG PET/MRI in asymptomatic adults were assessed. The uptake values reported in this study could serve as a reference for the identification of abnormalities in patients with chronic low back pain, but the applied PET reconstruction method and patient BMI should be taken into account.

## Introduction

Identification of the cause of chronic low back pain (LBP) is a challenging task, since both clinical examination and anatomical imaging often have limited value [1,2]. In recent years, molecular imaging techniques have gained interest for the detection of the source of pain. PET with the glucose analogue [^18^F]fluorodeoxyglucose ([^18^F]FDG) is a highly sensitive imaging technique that is increasingly applied in inflammatory disease [3]. Chronic pain is often accompanied by low-grade inflammation, which can be identified when FDG PET uptake is scaled more sensitively [4–7]. Accurate structural imaging remains essential for anatomical localization of the increased uptake. PET computed tomography (PET/CT) is the most often used hybrid imaging modality. More recently, hybrid PET–MRI (PET/MRI) systems have also become available. For spine imaging, PET/MRI has several important benefits, such as improved soft-tissue contrast and the ability to acquire different tissue contrasts, for example, for neurography. Several studies have shown promising results for identification of spinal pain generators with [^18^F]FDG PET/MRI [5–9].

Despite this promise, identification of spinal pain generators can still be challenging because of the subtle increased [^18^F]FDG uptake that sometimes just exceeds background or physiological uptake. To be able to adequately identify pain generators, it is important to understand the physiological [^18^F]FDG uptake values in spinal structures. Although this is relatively well known for spinal structures that are often involved in oncologic or infectious disease, such as vertebral bodies [10] and the spinal cord [11], few studies have reported on normal [^18^F]FDG uptake values in other spinal structures such as nerve roots, intervertebral discs, facet joints, or interspinous ligaments.

With quantitative PET, standardized uptake values (SUVs) are determined by correcting for patient size and the amount of injected radiotracer. This allows for comparison between exams or with a reference cohort, although scanner characteristics, acquisition and reconstruction protocols, and individual patient characteristics are known to induce variability in SUV measurements [12]. The use of within-patient SUV ratios with other tissues, such as the liver or subcutaneous fat, has been proposed as a method to overcome these problems with quantitative PET imaging [13].

The aim of this study is to evaluate physiological [^18^F]FDG uptake values in lumbar spine structures on PET/MRI in patients without LBP. Additional aims include comparing SUV metrics between commonly used reconstruction methods, explore within-patient SUV ratios with several reference tissues, and evaluate the effect of age, BMI, and blood glucose level on SUV metrics.

## Methods

### Study population

The Erasmus MC institutional research review board reviewed this study (MEC-2023-0184), and deemed it exempt from the Dutch Medical Research Involving Human Subjects Act. All participants provided written informed consent. Adult patients who were scheduled for [^18^F]FDG PET/MRI between July 2023 and March 2025 were potentially eligible. Only patients with nononcological indications were considered, since the spine is a common site for metastasis. Patients with known spinal abnormalities were also excluded. Directly after PET/MR examination, participants were asked to indicate if they had experienced pain in their low back or hip in the past week or during the examination using a numeric rating scale (NRS: 0–10). Participants indicating an NRS of greater than 2 were excluded from analysis. Other exclusion criteria were blood glucose level greater than 7.0 mmol/l, failed or incomplete image acquisition, excessive motion during acquisition, or poor coregistration between PET and MRI.

### Image acquisition and reconstruction

PET/MR images were acquired on a 3T SIGNA PET/MR system (GE Healthcare, Waukesha, Wisconsin, USA). Patients fasted for at least 6 h and participants’ height, weight, and blood glucose level were measured. Image acquisition started 55–70 min after intravenous injection of 0.033 MBq/kg^2^ (0.89 µCi/kg^2^) [^18^F]FDG. The PET imaging protocol consisted of six–eight bed positions from head to mid-femoral level at 3 min per bed position. T1-weighted Dixon magnetic resonance (MR) images for anatomical correlation were acquired simultaneously with PET emission data at each bed position, with repetition time 4.37 s, echo time 1.67, slice thickness 3 mm, and field-of-view 480 × 480 mm. For each bed position, dedicated T1-weighted Dixon images were acquired for attenuation correction with repetition time 4.0, echo time 1.67, slice thickness 5.2 mm, and field-of-view 500 × 500 mm.

PET emission data were corrected for random coincidences, dead time, and scatter. Attenuation correction was done using the standardized method used in clinical practice consisting of automated tissue segmentation and classification on the dedicated MR images. PET images were reconstructed at a voxel size of 2.34 × 2.34 × 2.78 mm^3^. PET images were reconstructed with an ordered subset expectation maximization (OSEM) algorithm incorporating point spread function and time-of-flight information with 28 subsets and four iterations, postfiltered with a 7.0 mm three-dimensional isotropic Gaussian filter. Additional PET image reconstructions were made with block sequential regularized expectation maximization (BSREM), incorporating point spread function and time-of-flight. Two BSREM reconstructions were made, one with regularization parameter *β* set to 300 and one with *β* value of 450.

### Image segmentation

**Fig. 1:**
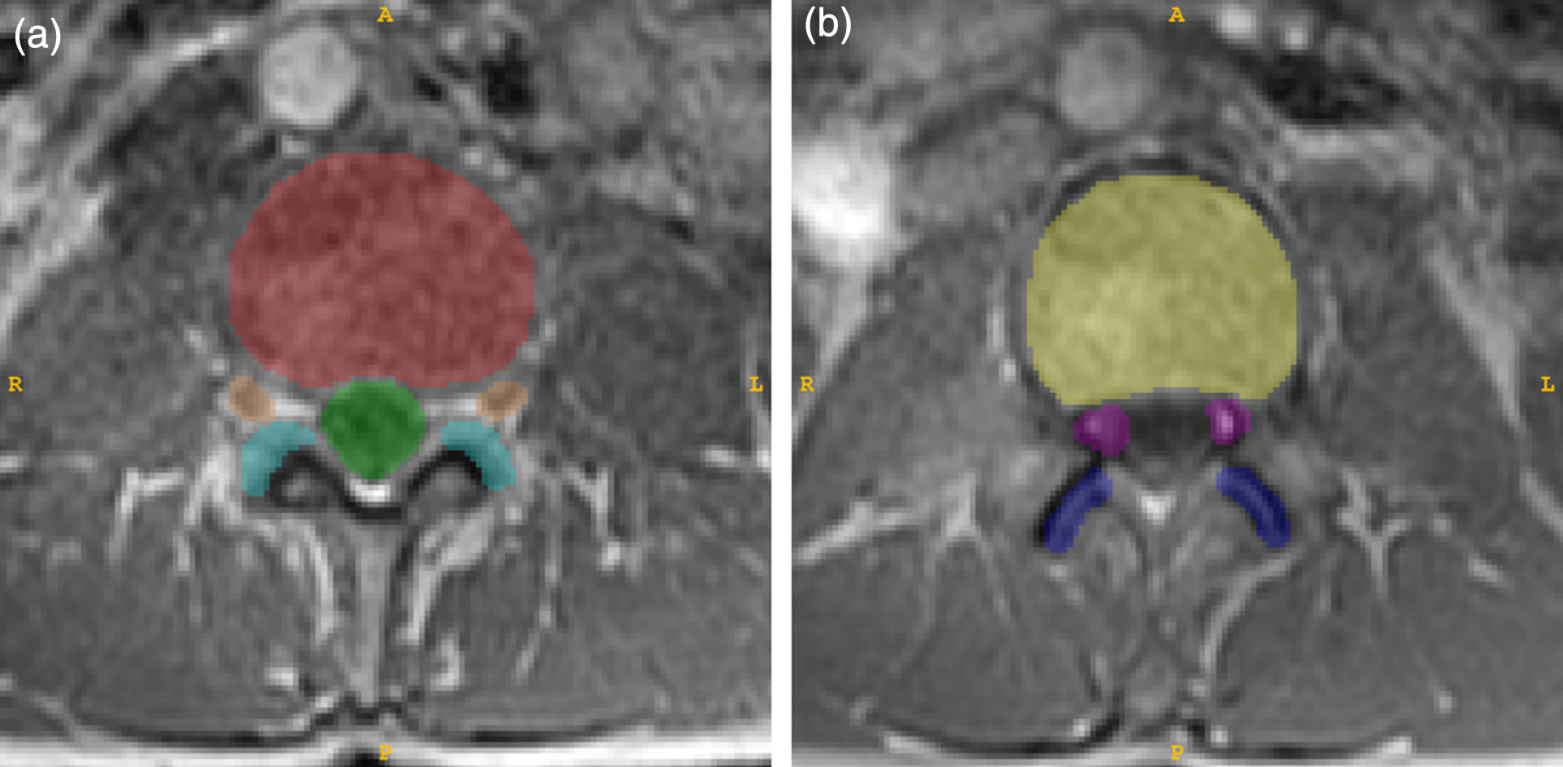
Example segmentations overlaid on the in-phase T1 weighted MRI. Images are cropped for viewing purposes. (a) Axial slice at the level of the intervertebral disc L3–L4, showing the disc (red), spinal canal (green), perifacet region (light blue), and nerve root (orange). (b) Axial slice at the level of vertebral body L3, showing the vertebral body (yellow), lateral recesses (purple), and facet joint (dark blue).

Three-dimensional volumes of interest (VOIs) in the lumbar spine were manually segmented on the T1-weighted Dixon MR images using ITK-SNAP software [14] by a trained researcher under supervision of a musculoskeletal radiologist with 5 years of experience. In-phase, fat, and water signal images were used to identify and segment structures. Care was taken to follow the anatomical borders of the respective structures. The following structures were included at spinal levels T12–S1: vertebral body, intervertebral disc, spinal canal at the level of the intervertebral disc, lateral recess, intraforaminal nerve root, facet joint, perifacet joint region, and the interspinous ligament. Example segmentations are shown in Fig. 1. In this study, intervertebral structures such as the intervertebral disc and interspinous ligament are referred to by both adjacent vertebral levels (e.g. L1–L2 for the structure in between vertebrae L1 and L2). Nerve roots and their corresponding lateral recesses are named after the vertebra above it, as is conventional in the lumbar spine [15]. Spherical reference VOIs of 1 cm^3^ were placed in the right liver lobe and in subcutaneous fat tissue in the right and left flanks.

PET images were converted to SUV by correcting for the injected tracer dose and patient bodyweight. All MRI derived VOIs were resampled to PET space and applied to the SUV-converted PET images to extract SUV_mean_ and SUV_max_ values from the OSEM, BSREM β300, and BSREM β450 images. The liver and subcutaneous fat VOIs were used to calculate normalized SUV_max_ (NSUV_max, liver_ and NSUV_max, subc_) and SUV_mean_ (NSUV_mean, liver_ and NSUV_mean, subc_) values.

### Potential to detect abnormalities

As an exploratory assessment, PET images of selected participants that reported LBP (NRS > 2) were evaluated by a PET trained musculoskeletal radiologist to assess areas showing abnormal [^18^F]FDG uptake. SUV values were scaled at 0–3 and visualized with the ‘rainbow’ color palette. SUV_max_ and SUV_mean_ were extracted for anatomical structures of interest.

### Statistics

Preliminary evaluation showed that SUV data was approximately normally distributed, so mean values and SDs across the study population were calculated for SUV_mean_ and SUV_max_ for all structures of interest. Mean values were calculated across spinal levels, as well as for each spinal level separately. Differences in SUV_max_ and SUV_mean_ between OSEM, BSREM β300, and BSREM β450 images were evaluated with repeated measures ANOVA. Correlations between SUV_max_ and age, BMI, and blood glucose level were evaluated by calculating the Pearson correlation coefficient. The effect of normalization on the variability of SUV_max_ and SUV_mean_ was evaluated using the coefficient of variation, calculated by dividing standard deviations by the mean values. *Z*-scores were calculated for the structures showing abnormal [^18^F]FDG uptake in the participants with pain. These *z*-scores indicate whether [^18^F]FDG uptake in the structures of interest was significantly higher than reference uptake values for the corresponding structures in asymptomatic participants. Bonferroni correction was applied to account for multiple comparison, and *P*-values less than 0.00625 were considered significant. All statistical analyses were performed using Python (version 3.12.9) and open-source statistics modules Pingouin (version 0.5.5) and Statsmodels (version 0.14.4) [16,17].

## Results

### Participants

Thirty patients were included in this study. From these, five patients were excluded from analysis because they reported pain scores greater than 2 in the low back before or during PET/MRI examination, and three patients were excluded because one of the PET reconstructions was missing. Of the remaining 22 participants, six were female (27.3%), mean age was 53.4 (SD: 12.5, range: 21–73) years, and mean BMI was 26.5 (SD: 4.6, range: 17.9–35.3) kg/m^2^. Median NRS for LBP was 0, with three participants reporting an NRS of 1 or 2 during imaging and four participants reporting an NRS of 1 or 2 in the past week.

### [^18^F]fluorodeoxyglucose uptake values in the lumbar spine

**Fig. 2:**
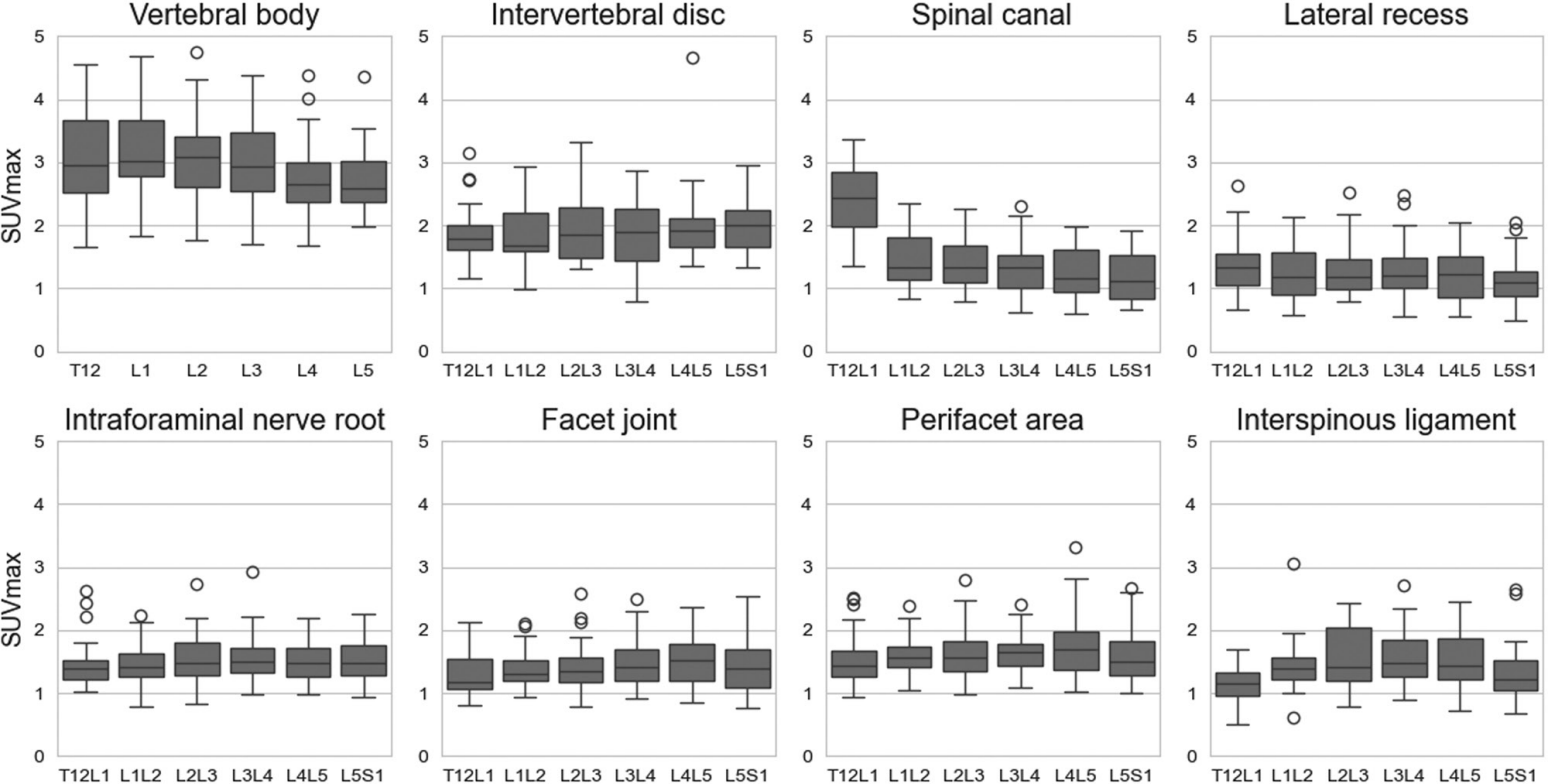
SUV_max_ in spinal structures at different levels in the lumbar spine on [^18^F]FDG PET/MRI in asymptomatic participants. PET images were reconstructed with OSEM. Boxplots show *Q*_1_, median, and *Q*_3_ values. [^18^F]FDG, [^18^F]fluorodeoxyglucose; OSEM, ordered subset expectation maximization; SUV_max_, maximum standardized uptake value.

Mean SUV_max_ on the OSEM PET reconstructions for all structures of interest and all lumbar spinal levels are presented in Table 1 and Fig. 2. SUV_mean_ values are presented in the Supplementary Table S1 (Supplemental digital content 1, https://links.lww.com/NMC/A408) and Supplementary Fig. S1 (Supplemental digital content 1, https://links.lww.com/NMC/A408). Of all structures in the lumbar spine, FDG uptake was highest in the vertebral body, with mean SUV_max_ 2.99 (SD: 0.69) and SUV_mean_ 1.60 (SD: 0.37). Intervertebral discs showed a mean SUV_max_ of 1.95 (SD: 0.54) and SUV_mean_ of 0.77 (SD: 0.24). In the nervous system, (peri-)articular structures, and ligamentous structures in the spine, FDG uptake was generally low, with SUV_max_ ranging between 1.26 and 1.62 and SUV_mean_ ranging between 0.76 and 1.02. FDG uptake was similar across the spinal levels for all structures, with the exception of the spinal canal, where both SUV_max_ and SUV_mean_ were higher at level T12–L1 compared to the other spinal levels. When the T12–L1 level was excluded, a mean SUV_max_ of 1.33 (SD: 0.43) and a mean SUV_mean_ of 0.79 (SD: 0.26) was found for the spinal canal.

**Table 1:** Maximum standardized uptake value [mean (SD)] in spinal structures at different levels in the lumbar spine on [^18^F]fluorodeoxyglucose PET/MRI with ordered subset expectation maximization reconstruction

Spinal level	Vertebral body	Intervertebral disc	Spinal canal	Lateral recess	Nerve root	Facet joint	Perifacet	Interspinous ligament
T12(–L1)	3.12 (0.77)	1.89 (0.49)	2.39 (0.61)	1.38 (0.40)	1.45 (0.33)	1.31 (0.37)	1.51 (0.39)	1.12 (0.29)
L1(–L2)	3.17 (0.69)	1.93 (0.51)	1.48 (0.47)	1.27 (0.42)	1.46 (0.34)	1.36 (0.27)	1.59 (0.30)	1.44 (0.46)
L2(–L3)	3.09 (0.69)	1.96 (0.54)	1.40 (0.42)	1.28 (0.40)	1.55 (0.40)	1.42 (0.34)	1.63 (0.40)	1.58 (0.50)
L3(–L4)	3.05 (0.67)	1.88 (0.57)	1.33 (0.43)	1.28 (0.41)	1.56 (0.36)	1.45 (0.37)	1.64 (0.31)	1.58 (0.44)
L4(–L5)	2.80 (0.66)	2.01 (0.68)	1.26 (0.42)	1.22 (0.41)	1.51 (0.33)	1.51 (0.39)	1.75 (0.47)	1.49 (0.46)
L5(–S1)	2.71 (0.57)	2.01 (0.43)	1.20 (0.41)	1.13 (0.38)	1.54 (0.35)	1.43 (0.41)	1.59 (0.42)	1.33 (0.50)

### Differences between PET reconstructions

Mean SUV_max_ across spinal levels on OSEM, BSREM β300, and BSREM β450 reconstructions are provided in Tables 2 and 3. Differences in SUV_mean_ across the three reconstructions were small but statistically significant. SUV_max_ values were significantly higher on BSREM β300 and lower on BSREM β450 compared to OSEM. Mean SUV_max_ and SUV_mean_ on the BSREM β300 and BSREM β450 reconstructions for all structures of interest and all lumbar spinal levels are provided in the Supplementary Tables S2–S5 (Supplemental digital content 1, https://links.lww.com/NMC/A408).

**Table 2:** Maximum standardized uptake value in structures of interest in the lumbar spine on [^18^F]fluorodeoxyglucose PET/MRI with ordered subset expectation maximization, block-subset regularized expectation maximization β300, and block-subset regularized expectation maximization β450 reconstructions. Values presented are mean (SD), calculated across all lumbar spinal levels

Structure	OSEM	BSREM β300	BSREM β450	*P* value
Vertebral body	2.99 (0.69)	3.29 (0.66)	2.79 (0.54)	<0.0001
Intervertebral disc	1.95 (0.54)	2.08 (0.54)	1.83 (0.47)	<0.0001
Spinal canal	1.51 (0.61)	1.59 (0.76)	1.37 (0.58)	<0.0001
Lateral recess	1.26 (0.41)	1.25 (0.38)	1.19 (0.34)	0.0022
Intraforaminal nerve root	1.51 (0.35)	1.54 (0.35)	1.38 (0.28)	<0.0001
Facet joint	1.41 (0.36)	1.44 (0.37)	1.27 (0.30)	<0.0001
Perifacet	1.62 (0.39)	1.75 (0.45)	1.51 (0.35)	<0.0001
Interspinous ligament	1.42 (0.47)	1.50 (0.55)	1.34 (0.46)	<0.0001

BSREM, block-subset regularized expectation maximization; OSEM, ordered subset expectation maximization.

**Table 3:** Mean standardized uptake value in structures of interest in the lumbar spine on [^18^F]fluorodeoxyglucose PET/MRI with ordered subset expectation maximization, block-subset regularized expectation maximization β300, and block-subset regularized expectation maximization β450 reconstructions. Values presented are mean (SD), calculated across all lumbar spinal levels

Structure	OSEM	BSREM β300	BSREM β450	*P* value
Vertebral body	1.60 (0.37)	1.61 (0.37)	1.60 (0.37)	<0.0001
Intervertebral disc	0.77 (0.24)	0.77 (0.25)	0.80 (0.25)	<0.0001
Spinal canal	0.88 (0.34)	0.86 (0.35)	0.86 (0.33)	<0.0001
Lateral recess	0.99 (0.30)	0.97 (0.30)	0.98 (0.28)	0.0108
Intraforaminal nerve root	1.02 (0.23)	1.02 (0.23)	1.01 (0.21)	0.0036
Facet joint	0.85 (0.21)	0.83 (0.21)	0.83 (0.20)	<0.0001
Perifacet	0.89 (0.19)	0.90 (0.19)	0.89 (0.18)	0.0023
Interspinous ligament	0.92 (0.25)	0.95 (0.28)	0.93 (0.26)	0.0002

BSREM, block-subset regularized expectation maximization; OSEM, ordered subset expectation maximization.

### Correlation with age, sex, and BMI

Pearson correlation coefficients for SUV_max_ with age, BMI, and blood glucose level are presented in Table 4. SUV_max_ was positively and significantly correlated with BMI for all structures of interest, with correlation coefficients ranging between 0.62 for the perifacet region and 0.82 for the nerve root. Correlation slopes ranged between 0.04 and 0.11, meaning that an increase in BMI of 1 kg/m^2^ results in a SUV_max_ increase of 0.04–0.11 on average. No correlations were found between SUV_max_ and age or blood glucose level.

**Table 4:** Pearson correlation coefficients between maximum standardized uptake value and patient characteristics

Structure	Age		BMI		Blood glucose	
	*r* (95% CI)	*P* value	*r* (95% CI)	*P* value	*r* (95% CI)	*P* value
Vertebral body	0.38 (−0.39, 0.45)	0.8676	0.81 (0.58, 0.92)	< 0.0001*	0.29 (−0.15, 0.63)	0.1951
Intervertebral disc	0.10 (−0.33, 0.50)	0.6521	0.73 (0.45, 0.88)	0.0001*	0.09 (−0.35, 0.49)	0.7049
Spinal canal	0.089 (−0.35, 0.49)	0.6946	0.72 (0.43, 0.88)	0.0001*	0.13 (−0.31, 0.52)	0.5587
Lateral recess	0.24 (−0.2, 0.60)	0.2822	0.80 (0.57, 0.91)	< 0.0001*	0.32 (−0.12, 0.65)	0.1509
Intraforaminal nerve root	0.23 (−0.21, 0.60)	0.2995	0.82 (0.60, 0.92)	< 0.0001*	0.51 (0.11, 0.77)	0.01500
Facet joint	0.06 (−0.37, 0.47)	0.7763	0.64 (0.30, 0.84)	0.0014*	0.39 (−0.04, 0.69)	0.07638
Perifacet	−0.04 (−0.45, 0.39)	0.8699	0.62 (0.27, 0.83)	0.0021*	0.14 (−0.30, 0.53)	0.5280
Interspinous ligament	0.08 (−0.35, 0.49)	0.7175	0.70 (0.39, 0.86)	0.0003*	0.09 (−0.35, 0.49)	0.6961

CI confidence interval.

* Statistically significant correlations.

### Normalized uptake values

Normalized uptake values NSUV_max, liver_ and NSUV_max, subcutaneous_ are presented in Supplementary Figs. S2–S5 (Supplemental digital content 1, https://links.lww.com/NMC/A408). Subcutaneous fat generally shows very low uptake and thus normalized SUV values against subcutaneous fat are high, while normalization against the liver results in values around or lower than 1. Coefficient of variations for NSUV_max, liver_ and NSUV_mean, liver_ (range: 0.21 – 0.39) were similar compared to non-normalized SUV_max_ and SUV_mean_ (range: 0.21–0.41). Coefficient of variation for NSUV_max, subcutaneous_ and NSUV_mean, subcutaneous_ was higher, ranging between 0.41 and 0.57.

### [^18^F]fluorodeoxyglucose uptake values in a patient with low back pain

**Fig. 3:**
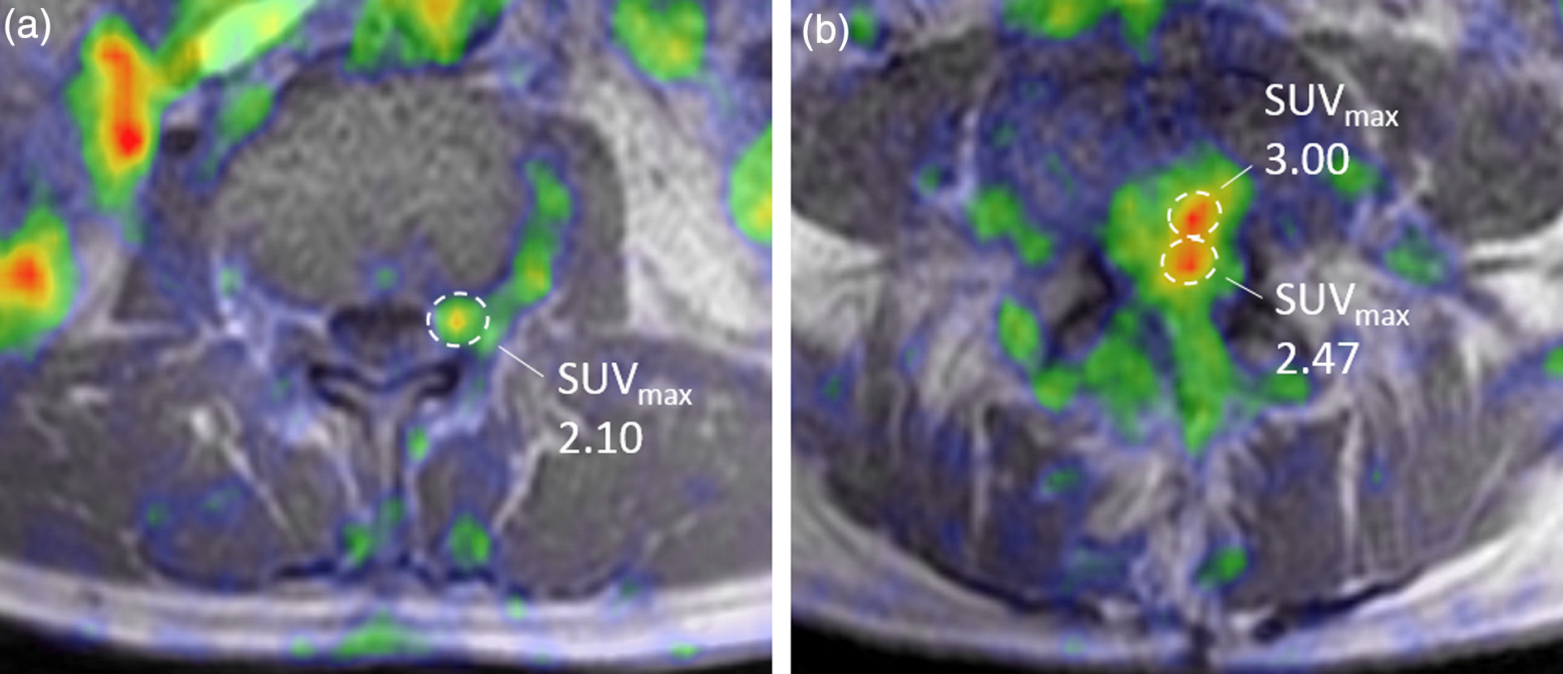
Example case of a patient with LBP, showing increased [^18^F]FDG uptake in two locations. (a) Increased uptake in the left lateral recess at level L2. (b) Abnormally increased uptake in a herniated intervertebral disc at level L4–L5, as well as the spinal canal directly posterior to the disc. [^18^F]FDG, [^18^F]fluorodeoxyglucose; LBP, low back pain; SUV_max_, maximum standardized uptake value.

We show an example cases of a 71-year-old male patient who reported an NRS of 7 for LBP at the time of PET/MR imaging and an average NRS of 5 in the week before the visit. Visual evaluation of the PET images showed focally increased [^18^F]FDG uptake in two locations (Fig. 3a and b). In the left lateral recess at level L2, SUV_max_ was 2.10 and SUV_mean_ was 1.43, and *z*-scores were 2.0 and 1.47 respectively. In the spinal canal posterior to intervertebral disc L4-L5, increased uptake was seen with SUV_max_ of 2.47 (*z*-score: 2.65) and SUV_mean_ of 1.87 (*z*-score: 4.54). MRI showed diffuse disc bulging and severe disc degeneration with bulging and ligamentum flavum hypertrophy at L4/5 and L5/S1. The affected intervertebral disc also showed focally increased [^18^F]FDG uptake with SUV_max_ of 3.00 (*z*-score: 1.43). Calculating *z*-scores for these regions using normalized SUV metrics resulted in slightly higher *z*-scores when normalized against the liver. For the lateral recess, *z*-scores were 2.53 for NSUV_max,liver_ and 2.02 for NSUV_mean,liver_ For the spinal canal, *z*-scores were 4.43 and 6.42 for NSUV_max,liver_ and NSUV_mean,liver_, respectively. Normalization against the subcutaneous fat for the same regions of interest resulted in *z*-scores between −0.53 and 0.64.

## Discussion

This study evaluated physiological [^18^F]FDG uptake values in the lumbar spine on PET/MRI. These findings could serve as a reference for the identification of abnormalities in the lumbar spine in patients with chronic LBP. Our results also indicate that the choice of PET reconstruction algorithm significantly affects SUV_max_ and to a lesser extent SUV_mean_ in the lumbar spine. As such, the appropriate reference values should be applied. Our findings indicate that SUV normalization against the liver or subcutaneous fat does not result in lower variability of SUV_max_ or SUV_mean_ in structures of the lumbar spine.

Previous studies have evaluated [^18^F]FDG uptake mainly on PET/CT, but some studies have shown that uptake values on PET/CT are significantly different from those on PET/MRI [18]. With the recent interest in [^18^F]FDG PET/MRI for chronic pain imaging, we believe that our reference values in confirmed pain-free individuals are a useful addition to the literature. Despite the technical and methodological differences between our study and others, our findings are generally in line with those reported for PET/CT. Normal [^18^F]FDG uptake in the vertebral body is driven by the relatively high metabolic activity of hematopoietic bone marrow. Nevertheless, SUV_max_ in the vertebral bone marrow as measured on PET/CT in healthy adults is typically lower than 3 [10]. In a direct comparison of [^18^F]FDG uptake on PET/CT and PET/MRI, Aiello *et al*. found a SUV_max_ of 3.73 (SD: 1.43) on PET/MRI in bone marrow at vertebral level L3, significantly higher than on PET/CT [18]. Some studies have indicated that bone marrow uptake is not uniformly distributed across spinal levels, but gradually decreases towards the cervical spine and towards the lower lumbar region [19]. Bone marrow uptake values are also reported to negatively correlate with age, especially in the lumbar region, as hematopoietic red marrow is replaced by yellow marrow [10]. However, we were unable to reproduce this finding in the current study.

Intervertebral discs generally do not show significant [^18^F]FDG uptake, and increased uptake can be indicative of underlying pathology such as spondylodiscitis [20,21]. The low-grade inflammation associated with disc degeneration may also increase [^18^F]FDG uptake, which could explain why patients with LBP have been shown to display slightly higher mean [^18^F]FDG uptake compared to healthy controls [22]. We found a relatively large difference between SUV_max_ and SUV_mean_ for the intervertebral disc. Although this could be because of normal heterogeneity in [^18^F]FDG uptake within the intervertebral disc, the high physiological uptake in the adjacent vertebral bone marrow also is a likely source of contamination that could contribute to this finding, and normalization against the bone marrow uptake may be beneficial.

Normal [^18^F]FDG uptake in the spinal canal has extensively been evaluated on PET/CT, with a large systematic review showing a decreasing trend in uptake from cervical to lumbar levels. In many patients, increased physiological uptake around level T11–L1 was seen [11,23]. Our study also indicated higher uptake values in the spinal canal at level L1. Although the exact mechanism is still unclear, this uptake pattern has been theorized to originate from the increased ratio of gray matter in the lumbar enlargement of the spinal cord, which houses the neuronal activity for the lower limbs [19]. Interestingly, a study evaluating spinal cord uptake in patients with LBP found significantly higher [^18^F]FDG uptake in the lower thoracic region for LBP patients compared to pain-free controls [24]. This increase in glucose metabolism driven by the neuronal activity associated with LBP could potentially be used to localize sources of LBP or guide treatment. Some studies suggest that [^18^F]FDG uptake in the spinal cord is often contaminated by the adjacent vertebral bone marrow, and that normalization against bone marrow uptake could improve spinal cord evaluation [25].

Our findings show that nerve roots in asymptomatic patients generally do not show any notable [^18^F]FDG uptake in the lateral recess or neuroforamen. One study showed that in patients with chronic pain resulting from intervertebral disc herniation, [^18^F]FDG uptake is significantly higher in impinged nerve roots compared to the contralateral nerve root and healthy controls [6]. Notably, nerve root SUV_max_ findings for asymptomatic controls in this study were lower than in our cohort. However, the PET reconstruction parameters are not reported and, therefore, a direct comparison is not possible.

Intra- or peri-articular [^18^F]FDG uptake is often seen in inflammatory joint diseases, such as rheumatoid arthritis or osteoarthritis [26]. Facet joint arthropathy may also show increased uptake on [^18^F]FDG PET [27]. Although facet joint [^18^F]FDG uptake is associated with osteoarthritic findings on MRI, concordance with clinical localization based on pain symptoms is low [28]. As such, [^18^F]FDG PET could potentially be used to guide treatment in patients with suspected facet arthropathy, but its value currently remains subject of investigation.

Finally, Baastrup’s disease is a common but underdiagnosed painful condition characterized by inflammation and hypertrophy of the interspinous ligaments, caused by bone-on-bone contact of adjacent spinous processes [29]. [^18^F]FDG PET has shown potential to accurately detect the associated intraspinous hypermetabolism in patients with Baastrup’s disease, but more research is needed to assess its value in clinical practice [30].

In our study cohort, BMI was significantly and positively correlated with [^18^F]FDG uptake of all structures. Prior studies have also found SUV overestimation for other organs in obese patients, and suggested that SUV normalization to lean body mass could be used to eliminate the effect confounding effect of BMI [31,32]. We found no significant correlation between [^18^F]FDG uptake and blood glucose level. Earlier studies also concluded that glycemia has negligible impact on SUV in most organs, and only the brain showed significantly lower uptake in hyperglycemia [33].

Although quantitative PET imaging has recently been gaining interest in the research domain, in clinical practice SUV thresholds are not widely accepted as a basis for diagnosis because of the large degree of variability in measured SUVs [12]. Both biological and technical sources of error contribute to this variability. Despite important steps towards PET standardization [34], measured SUVs can significantly differ between scanners because of differences in detector sensitivity, acquisition parameters, and image reconstruction techniques [35–37]. Comparison between PET/CT and PET/MRI is especially problematic, since image reconstruction methods in PET/MRI rely on MRI-based attenuation correction methods that do not incorporate attenuation by bone [38]. This inability to account for bone attenuation can lead to large biases in SUV on PET/MRI, especially in or near bony structures such as the spinal column [39–41].

Normalization of SUV has been proposed as a method to pool or compare PET data across scanners by correcting for scanner sensitivity. The liver is most often used as the refence organ of choice [42,43]. However, the liver may not always be in the field of view for dedicated lumbar spine PET examination. Subcutaneous fat may provide a robust alternative reference value for SUV normalization. Our findings indicate that normalization of SUV against internal reference values in both the liver and subcutaneous fat does not result in a lower coefficient of variation compared to non-normalized SUV in this single-center study. Nevertheless, normalized SUV metrics may be valuable for generalizing the values presented in this study to other sites, even when acquisition and reconstruction protocols are harmonized.

A major strength of our study is the prospective design, which allowed us to only include confirmed asymptomatic participants, which contributes to the validity of our findings. Although in this study we aimed to establish reference values that can be used for LBP imaging, this reference may also be useful in case of other nononcological abnormalities in the spine. Some limitations of our study need to be taken into account. Firstl, we did not control for factors that may affect [^18^F]FDG uptake as reported in some prior studies, such as lean body mass or body surface area. Second, we also did not correct uptake values in the spinal canal or intervertebral discs for possible contamination by the adjacent bone marrow activity, as was suggested in some earlier studies. Instead, we chose to present our findings as is, allowing for direct comparison of uptake values with those in the other spinal structures. It is also important to note that the uptake values presented in this study were determined on PET/MRI, and as mentioned earlier these findings likely cannot be translated directly to PET/CT cases. Another potential limitation is the use of a whole-body MRI protocol with relatively low spatial resolution. A dedicated spine MRI protocol would have allowed for easier anatomical delineation of the spinal structures but would also require significantly longer acquisition times. Considering the inherently low resolution of PET imaging, we consider our current MRI protocol of adequate quality for this study. Moreover, delineation was supervised by an experienced musculoskeletal radiologist. Another important limitation with respect to the low spatial resolution of PET imaging is its sensitivity to partial volume effects [44]. Partial volume effects can result in a significant underestimation of SUV metrics in smaller regions of interest. The minimum region size needed to avoid most of the partial volume effects is tied to intrinsic scanner characteristics and generally is around 1 cm in region diameter. That means that for the smaller anatomical structures such as the nerve roots, our SUV measurements likely underestimate the actual uptake values. The parameter SUV_mean_ is generally more affected by partial voluming than SUV_max_ [44]. Although both SUV_mean_ and SUV_max_ have their own challenges, they are still widely used in clinical practice and, therefore, both shown in this paper. Finally, based on ethical considerations, no longitudinal follow-up in terms of pain score or treatment outcome was performed for the LBP patient that we presented in this study. However, we present this case only to illustrate how our reference values could be applied for the identification of painful spinal abnormalities.

To conclude, in this article, physiological [^18^F]FDG PET/MRI uptake values in the lumbar spine structures are presented for several clinically used PET reconstruction methods. These uptake values could serve as a reference for the identification of abnormalities in patients with chronic LBP, but the applied PET reconstruction method, patient BMI, and most importantly the patient specific complaints and any other concurrent imaging findings should be taken into account.

## Acknowledgements

J.M.M., R.A.v.d.H, and E.H.G.O. contributed to the study conception and design. Data collection and analysis were performed by J.M.M., D.W.J.v.B., and R. A.v.d.H. The first draft of the manuscript was written by J.M.M. and all authors commented on previous versions of the manuscript. All authors read and approved the final manuscript.

This study was performed in line with the principles of the Declaration of Helsinki and local and national laws and guidelines. The Erasmus MC institutional research review board reviewed this study (MEC-2023-0184), and deemed it exempt from the Dutch Medical Research Involving Human Subjects Act. Informed consent was obtained from all individual participants included in the study. Participants provided informed consent for publication of their images.

### Conflicts of interest

E.H.G.O. has received research grants from GE Healthcare. For the remaining authors, there are no conflicts of interest.

## Supplementary Material



## References

[1] Chou R Fu R Carrino JA Deyo RA. Imaging strategies for low-back pain: systematic review and meta-analysis. Lancet 2009; 373:463–472. 19200918 10.1016/S0140-6736(09)60172-0

[2] Eloqayli H. Clinical decision-making in chronic spine pain: dilemma of image-based diagnosis of degenerative spine and generation mechanisms for nociceptive, radicular, and referred pain. Biomed Res Int 2018; 2018:1–6. 10.1155/2018/8793843 PMC6311773 30648110

[3] Katal S Gholamrezanezhad A Nikpanah M Christensen TQ Werner TJ Saboury B. Potential applications of PET/CT/MR imaging in inflammatory diseases: part I: musculoskeletal and gastrointestinal systems. PET Clin 2020; 15:547–558. 32768367 10.1016/j.cpet.2020.06.008

[4] Yoon D Kogan F Gold G Biswal S. Identifying musculoskeletal pain generators using molecular imaging. In: Ross BD, Gambhir SS, editors. Molecular Imaging (Second Edition). Academic Press; 2021. pp. 1373–1392.

[5] Biswal S Behera D Yoon DH Holley D Ith MAM Carroll I. [18F]FDG PET/MRI of patients with chronic pain alters management: early experience. EJNMMI Phys 2015; 2:A84. 26956346 10.1186/2197-7364-2-S1-A84 PMC4798651

[6] Cipriano PW Yoon D Gandhi H Holley D Thakur D Hargreaves BA. 18F-FDG PET/MRI in chronic sciatica: early results revealing spinal and nonspinal abnormalities. J Nucl Med 2018; 59:967–972. 29097408 10.2967/jnumed.117.198259

[7] Weng Y-S Tang C-T Chang W-C Huang G-S Chiu C-H Chiang S-W. Added value of 18Fluorine-fluorodeoxyglucose (18F-FDG) PET/MRI for evaluation of failed back surgery syndrome: comparison with non-contrast MRI. Jpn J Radiol 2025; 43:509–519. 39404924 10.1007/s11604-024-01679-0

[8] Mostert J Oei E Ananta M Muradin G Huygen F de Vos C. Hybrid positron emission tomography and MRI (PET-MRI) with [18F]FDG for identifying musculoskeletal pain generators: early experience. ISMRM Annu Meet 2024.

[9] Pirasteh A Reddy V Biswal S. 18F-FDG PET/MRI alters management or increases diagnostic confidence in patients with challenging spinal and lower extremity pain: preliminary experience in 12 patients. J Nucl Med 2024; 65:242537.

[10] Shen G Liang M Su M Kuang A. Physiological uptake of 18F-FDG in the vertebral bone marrow in healthy adults on PET/CT imaging. Acta Radiol 2018; 59:1487–1493. 29486597 10.1177/0284185118762245

[11] Kiamanesh Z Banezhad F Nasiri Z Emami F Treglia G Sadeghi R. Physiological distribution of 18F-FDG in the spinal cord: a systematic review. J Spinal Cord Med 2021; 44:517–524. 31682787 10.1080/10790268.2019.1672954 PMC8288118

[12] Kinahan PE Fletcher JW. Positron emission tomography-computed tomography standardized uptake values in clinical practice and assessing response to therapy. Semin Ultrasound CT MR 2010; 31:496–505. 21147377 10.1053/j.sult.2010.10.001 PMC3026294

[13] Sarikaya I Sarikaya A. Assessing PET parameters in oncologic 18F-FDG studies. J Nucl Med Technol 2020; 48:278–282. 31811061 10.2967/jnmt.119.236109

[14] Yushkevich PA Piven J Hazlett HC Smith RG Ho S Gee JC Gerig G. User-guided 3D active contour segmentation of anatomical structures: significantly improved efficiency and reliability. Neuroimage 2006; 31:1116–1128. 16545965 10.1016/j.neuroimage.2006.01.015

[15] Kaiser JT Lugo-Pico JG. Neuroanatomy, spinal nerves. StatPearls. StatPearls Publishing; 2025. http://www.ncbi.nlm.nih.gov/books/NBK542218/. [Accessed 18 July 2025] 31194375

[16] Vallat R. Pingouin: statistics in Python. JOSS 2018; 3:1026.

[17] Seabold S Perktold J. Statsmodels: econometric and statistical modeling with Python. SciPy; 2010. p. 92–96. 10.25080/Majora-92bf1922-011. [Accessed 30 July 2025]

[18] Aiello M Alfano V Salvatore E Cavaliere C Picardi M Della Pepa R. [18F]FDG uptake of the normal spinal cord in PET/MR imaging: comparison with PET/CT imaging. EJNMMI Res 2020; 10:91. 32761394 10.1186/s13550-020-00680-8 PMC7410944

[19] Patel PY Dalal I Griffith B. [18F]FDG-PET evaluation of spinal pathology in patients in oncology: pearls and pitfalls for the neuroradiologist. AJNR Am J Neuroradiol 2022; 43:332–340. 34711547 10.3174/ajnr.A7308 PMC8910786

[20] Hungenbach S Delank K-S Dietlein M Eysel P Drzezga A Schmidt MC. 18F-fluorodeoxyglucose uptake pattern in patients with suspected spondylodiscitis. Nucl Med Commun 2013; 34:1068–1074. 24025917 10.1097/MNM.0b013e328365abec

[21] Lazzeri E Bozzao A Cataldo MA Petrosillo N Manfrè L Trampuz A. Joint EANM/ESNR and ESCMID-endorsed consensus document for the diagnosis of spine infection (spondylodiscitis) in adults. Eur J Nucl Med Mol Imaging 2019; 46:2464–2487. 31399800 10.1007/s00259-019-04393-6

[22] Piri R Nøddeskou‐Fink AH Gerke O Larsson M Edenbrandt L Enqvist O. PET/CT imaging of spinal inflammation and microcalcification in patients with low back pain: a pilot study on the quantification by artificial intelligence‐based segmentation. Clin Physio Funct Imaging 2022; 42:225–232. 10.1111/cpf.12751 PMC9322590 35319166

[23] Do BH Mari C Tseng JR Quon A Rosenberg J Biswal S. Pattern of 18F-FDG uptake in the spinal cord in patients with non-central nervous system malignancy. Spine (Phila Pa 1976) 2011; 36:E1395–E1401. 21311407 10.1097/BRS.0b013e31820a7df8

[24] Zhou X Cipriano P Kim B Dhatt H Rosenberg J Mittra E. Detection of nociceptive-related metabolic activity in the spinal cord of low back pain patients using 18F-FDG PET/CT. Scand J Pain 2017; 15:53–57. 28850345 10.1016/j.sjpain.2016.11.017

[25] Patel NJ Gupta V Vibhute PG Jain MK Accurso JM. A large cohort study of 18f fluoro-deoxy-glucose uptake in normal spinal cord: quantitative assessment of the contamination from adjacent vertebral marrow uptake and validity of normalizing the cord uptake against the lumbar thecal sac. J Comput Assist Tomogr 2017; 41:125–130. 27560019 10.1097/RCT.0000000000000479

[26] Kogan F Yoon D Teeter MG Chaudhari AJ Hales L Barbieri M. Multimodal positron emission tomography (PET) imaging in non-oncologic musculoskeletal radiology. Skeletal Radiol 2024; 53:1833–1846. 38492029 10.1007/s00256-024-04640-4 PMC12778996

[27] Rosen RS Fayad L Wahl RL. Increased 18F-FDG uptake in degenerative disease of the spine: characterization with 18F-FDG PET/CT. J Nucl Med 2006; 47:1274–1280. 16883005

[28] Lehman VT Diehn FE Broski SM Nathan MA Kemp BJ Larson NB. Comparison of [18 F] FDG-PET/MRI and clinical findings for assessment of suspected lumbar facet joint pain: a prospective study to characterize candidate nonanatomic imaging biomarkers and potential impact on management. Am J Neuroradiol 2019; 40:1779–1785. 31558502 10.3174/ajnr.A6224 PMC7028568

[29] Philipp LR Baum GR Grossberg JA Ahmad FU. Baastrup’s disease: an often missed etiology for back pain. Cureus 2016; 8:e465. 26929892 10.7759/cureus.465 PMC4762769

[30] Piracha AZ Suthar PP Virmani S. 18F-Fluorodeoxyglucose (18F-FDG) PET/CT appearance of baastrup’s disease and its multimodality correlation. Cureus 2024; 16:e64093. 39114245 10.7759/cureus.64093 PMC11305603

[31] Sarikaya I Albatineh AN Sarikaya A. Revisiting weight-normalized suv and lean-body-mass-normalized SUV in PET studies. J Nucl Med Technol 2020; 48:163–167. 31604893 10.2967/jnmt.119.233353

[32] Sharma P Chatterjee P Alvarado LA Dwivedi AK. Standardized uptake value of normal organs on routine clinical [18F]FDG PET/CT: impact of tumor metabolism and patient-related factors. Nucl. Med. Rev. 2022. 10.5603/NMR.a2022.0036 36286203

[33] Sprinz C Altmayer S Zanon M Watte G Irion K Marchiori E Hochhegger B. Effects of blood glucose level on 18F-FDG uptake for PET/CT in normal organs: a systematic review. PLoS One 2018; 13:e0193140. 29486008 10.1371/journal.pone.0193140 PMC5828444

[34] Aide N Lasnon C Veit-Haibach P Sera T Sattler B Boellaard R. EANM/EARL harmonization strategies in PET quantification: from daily practice to multicentre oncological studies. Eur J Nucl Med Mol Imaging 2017; 44:17–31. 10.1007/s00259-017-3740-2 PMC5541084 28623376

[35] Boellaard R. Standards for PET image acquisition and quantitative data analysis. J Nucl Med 2009; 50:11S–20S. 19380405 10.2967/jnumed.108.057182

[36] Teoh EJ McGowan DR Macpherson RE Bradley KM Gleeson FV. Phantom and clinical evaluation of the bayesian penalized likelihood reconstruction algorithm Q.Clear on an LYSO PET/CT system. J Nucl Med 2015; 56:1447–1452. 26159585 10.2967/jnumed.115.159301 PMC4558942

[37] Matti A Lima GM Pettinato C Pietrobon F Martinelli F Fanti S. How do the more recent reconstruction algorithms affect the interpretation criteria of PET/CT images? Nucl Med Mol Imaging 2019; 53:216–222. 31231442 10.1007/s13139-019-00594-x PMC6554360

[38] Catana C. Attenuation correction for human PET/MRI studies. Phys Med Biol 2020; 65:23TR02. 10.1088/1361-6560/abb0f8 PMC8590198 33263313

[39] Izquierdo-Garcia D Catana C. MR imaging-guided attenuation correction of PET data in PET/MR imaging. PET Clin 2016; 11:129–149. 26952727 10.1016/j.cpet.2015.10.002 PMC5798860

[40] Brancato V Borrelli P Alfano V Picardi M Mascalchi M Nicolai E. The impact of MR-based attenuation correction in spinal cord FDG-PET/MR imaging for neurological studies. Med Phys 2021; 48:5924–5934. 34369590 10.1002/mp.15149 PMC9293017

[41] Samarin A Burger C Wollenweber SD Crook DW Burger IA Schmid DT. PET/MR imaging of bone lesions--implications for PET quantification from imperfect attenuation correction. Eur J Nucl Med Mol Imaging 2012; 39:1154–1160. 22526955 10.1007/s00259-012-2113-0

[42] Paquet N Albert A Foidart J Hustinx R. Within-patient variability of (18)F-FDG: standardized uptake values in normal tissues. J Nucl Med 2004; 45:784–788. 15136627

[43] Kuhnert G Boellaard R Sterzer S Kahraman D Scheffler M Wolf J. Impact of PET/CT image reconstruction methods and liver uptake normalization strategies on quantitative image analysis. Eur J Nucl Med Mol Imaging 2016; 43:249–258. 26280981 10.1007/s00259-015-3165-8

[44] Soret M Bacharach SL Buvat I. Partial-volume effect in PET tumor imaging. J Nucl Med 2007; 48:932–945. 17504879 10.2967/jnumed.106.035774

