# Supplementary material for: [18F]fluorodeoxyglucose PET/MRI for nononcological musculoskeletal indications: reference uptake values in the asymptomatic lumbar spine and example comparison to a low back pain patient
Source: Nucl Med Commun. 2026 Apr 27;47(9):1072–80. doi: 10.1097/MNM.0000000000002164 (PMC13440533; doi:10.1097/MNM.0000000000002164)
Supplement: Supplementary file 1 [file nmc-47-1072-s001.docx]

Supplemental digital content to:

**[^18^F]FDG PET/MRI for non-oncological musculoskeletal indications: reference uptake values in the asymptomatic lumbar spine and example comparison to a low back pain patient**

Table S1. Mean SUV_mean_ (SD) in structures of interest at different spinal levels in the lumbar spine on [^18^F]FDG PET/MRI with OSEM reconstruction.

|  | **Vertebral body** | **Intervertebral disc** | **Spinal canal** | **Lateral recess** | **Nerve root** | **Facet joint** | **Perifacet** | **Interspinous ligament** |
| --- | --- | --- | --- | --- | --- | --- | --- | --- |
| **T12(-L1)** | 1.68 (0.38) | 0.78 (0.25) | 1.35 (0.34) | 1.09 (0.31) | 0.99 (0.21) | 0.80 (0.21) | 0.84 (0.19) | 0.74 (0.16) |
| **L1(-L2)** | 1.68 (0.37) | 0.75 (0.22) | 0.88 (0.23) | 1.01 (0.30) | 1.03 (0.20) | 0.84 (0.16) | 0.90 (0.16) | 0.90 (0.22) |
| **L2(-L3)** | 1.68 (0.41) | 0.76 (0.21) | 0.85 (0.26) | 1.00 (0.29) | 1.05 (0.23) | 0.87 (0.20) | 0.92 (0.16) | 1.02 (0.27) |
| **L3(-L4)** | 1.62 (0.35) | 0.71 (0.23) | 0.79 (0.24) | 0.97 (0.30) | 1.05 (0.22) | 0.90 (0.22) | 0.94 (0.19) | 1.04 (0.23) |
| **L4(-L5)** | 1.54 (0.33) | 0.74 (0.31) | 0.74 (0.27) | 0.96 (0.31) | 1.03 (0.24) | 0.87 (0.20) | 0.93 (0.20) | 1.01 (0.26) |
| **L5(-S1)** | 1.41 (0.34) | 0.85 (0.23) | 0.67 (0.23) | 0.90 (0.29) | 0.97 (0.25) | 0.79 (0.21) | 0.82 (0.19) | 0.83 (0.23) |

Figure S1. Mean SUV_mean_ (SD) in spinal structures at different levels in the lumbar spine on [^18^F]FDG PET/MRI with OSEM reconstruction.

Table S2. Mean SUV_max_ (SD) in structures of interest at different spinal levels in the lumbar spine on [^18^F]FDG PET with BSREM β300 reconstruction.

|  | **Vertebral body** | **Intervertebral disc** | **Spinal canal** | **Lateral recess** | **Nerve root** | **Facet joint** | **Perifacet** | **Interspinous ligament** |
| --- | --- | --- | --- | --- | --- | --- | --- | --- |
| **T12(-L1)** | 3.29 (0.57) | 1.99 (0.45) | 2.84 (0.92) | 1.35 (0.36) | 1.41 (0.28) | 1.26 (0.38) | 1.54 (0.41) | 1.09 (0.31) |
| **L1(-L2)** | 3.49 (0.72) | 2.04 (0.50) | 1.49 (0.44) | 1.23 (0.39) | 1.46 (0.35) | 1.40 (0.34) | 1.72 (0.44) | 1.52 (0.62) |
| **L2(-L3)** | 3.27 (0.60) | 2.16 (0.45) | 1.38 (0.37) | 1.26 (0.36) | 1.58 (0.40) | 1.44 (0.32) | 1.74 (0.39) | 1.73 (0.61) |
| **L3(-L4)** | 3.49 (0.74) | 1.96 (0.55) | 1.34 (0.37) | 1.26 (0.35) | 1.63 (0.38) | 1.47 (0.36) | 1.82 (0.36) | 1.70 (0.56) |
| **L4(-L5)** | 3.22 (0.63) | 2.24 (0.82) | 1.30 (0.39) | 1.24 (0.44) | 1.60 (0.33) | 1.55 (0.36) | 1.97 (0.52) | 1.55 (0.44) |
| **L5(-S1)** | 2.98 (0.57) | 2.08 (0.35) | 1.19 (0.39) | 1.16 (0.41) | 1.57 (0.32) | 1.50 (0.40) | 1.70 (0.49) | 1.41 (0.51) |

Table S3. Mean SUV_mean_ (SD) in structures of interest at different spinal levels in the lumbar spine on [^18^F]FDG PET with BSREM β300 reconstruction.

|  | **Vertebral body** | **Intervertebral disc** | **Spinal canal** | **Lateral recess** | **Nerve root** | **Facet joint** | **Perifacet** | **Interspinous ligament** |
| --- | --- | --- | --- | --- | --- | --- | --- | --- |
| **T12(-L1)** | 1.69 (0.38) | 0.80 (0.26) | 1.37 (0.35) | 1.08 (0.30) | 0.98 (0.21) | 0.76 (0.21) | 0.84 (0.19) | 0.73 (0.19) |
| **L1(-L2)** | 1.70 (0.37) | 0.76 (0.24) | 0.86 (0.23) | 1.00 (0.31) | 1.04 (0.20) | 0.83 (0.18) | 0.91 (0.16) | 0.93 (0.23) |
| **L2(-L3)** | 1.69 (0.41) | 0.76 (0.23) | 0.82 (0.26) | 0.97 (0.26) | 1.06 (0.23) | 0.85 (0.20) | 0.92 (0.16) | 1.08 (0.31) |
| **L3(-L4)** | 1.64 (0.36) | 0.70 (0.25) | 0.76 (0.25) | 0.95 (0.27) | 1.06 (0.22) | 0.88 (0.23) | 0.95 (0.19) | 1.06 (0.25) |
| **L4(-L5)** | 1.56 (0.33) | 0.73 (0.31) | 0.72 (0.28) | 0.93 (0.33) | 1.03 (0.23) | 0.85 (0.20) | 0.94 (0.20) | 1.04 (0.26) |
| **L5(-S1)** | 1.41 (0.34) | 0.84 (0.23) | 0.65 (0.23) | 0.89 (0.29) | 0.97 (0.25) | 0.78 (0.22) | 0.82 (0.19) | 0.86 (0.24) |

Table S4. Mean SUV_max_ (SD) in structures of interest at different spinal levels in the lumbar spine on [^18^F]FDG PET with BSREM β450 reconstruction.

|  | **Vertebral body** | **Intervertebral disc** | **Spinal canal** | **Lateral recess** | **Nerve root** | **Facet joint** | **Perifacet** | **Interspinous ligament** |
| --- | --- | --- | --- | --- | --- | --- | --- | --- |
| **T12(-L1)** | 2.82 (0.51) | 1.74 (0.39) | 2.33 (0.66) | 1.32 (0.32) | 1.32 (0.26) | 1.13 (0.30) | 1.35 (0.36) | 1.01 (0.26) |
| **L1(-L2)** | 2.89 (0.55) | 1.78 (0.44) | 1.28 (0.31) | 1.21 (0.34) | 1.32 (0.26) | 1.22 (0.27) | 1.49 (0.33) | 1.32 (0.54) |
| **L2(-L3)** | 2.85 (0.56) | 1.90 (0.35) | 1.21 (0.28) | 1.21 (0.33) | 1.42 (0.32) | 1.27 (0.26) | 1.48 (0.28) | 1.50 (0.46) |
| **L3(-L4)** | 2.91 (0.56) | 1.71 (0.44) | 1.16 (0.30) | 1.20 (0.31) | 1.43 (0.29) | 1.30 (0.29) | 1.55 (0.27) | 1.52 (0.47) |
| **L4(-L5)** | 2.74 (0.57) | 1.97 (0.74) | 1.15 (0.34) | 1.16 (0.37) | 1.43 (0.28) | 1.38 (0.30) | 1.65 (0.41) | 1.41 (0.39) |
| **L5(-S1)** | 2.51 (0.48) | 1.86 (0.36) | 1.06 (0.31) | 1.04 (0.32) | 1.39 (0.29) | 1.29 (0.35) | 1.47 (0.38) | 1.26 (0.42) |

Table S5. Mean SUV_mean_ (SD) in structures of interest at different spinal levels in the lumbar spine on [^18^F]FDG PET with BSREM β450 reconstruction.

|  | **Vertebral body** | **Intervertebral disc** | **Spinal canal** | **Lateral recess** | **Nerve root** | **Facet joint** | **Perifacet** | **Interspinous ligament** |
| --- | --- | --- | --- | --- | --- | --- | --- | --- |
| **T12(-L1)** | 1.67 (0.37) | 0.84 (0.26) | 1.32 (0.33) | 1.10 (0.28) | 0.97 (0.20) | 0.77 (0.19) | 0.83 (0.17) | 0.73 (0.16) |
| **L1(-L2)** | 1.67 (0.36) | 0.80 (0.24) | 0.85 (0.21) | 1.02 (0.28) | 1.02 (0.17) | 0.83 (0.16) | 0.90 (0.15) | 0.91 (0.22) |
| **L2(-L3)** | 1.67 (0.40) | 0.79 (0.23) | 0.82 (0.24) | 0.99 (0.26) | 1.04 (0.21) | 0.86 (0.19) | 0.91 (0.15) | 1.05 (0.27) |
| **L3(-L4)** | 1.62 (0.36) | 0.73 (0.25) | 0.76 (0.24) | 0.96 (0.25) | 1.03 (0.21) | 0.89 (0.21) | 0.94 (0.18) | 1.04 (0.24) |
| **L4(-L5)** | 1.55 (0.33) | 0.76 (0.31) | 0.74 (0.27) | 0.94 (0.30) | 1.02 (0.23) | 0.86 (0.20) | 0.93 (0.19) | 1.03 (0.26) |
| **L5(-S1)** | 1.40 (0.33) | 0.86 (0.23) | 0.66 (0.22) | 0.87 (0.26) | 0.96 (0.24) | 0.78 (0.21) | 0.82 (0.18) | 0.84 (0.22) |

Figure S2. SUV_max_ normalized against the liver (NSUV_max, liver_) for spinal structures at different levels in the lumbar spine. PET images were reconstructed with OSEM. Boxplots show Q_1_, median, and Q_3_ values.

Figure S3. SUV_mean_ normalized against the liver (NSUV_mean, liver_) for spinal structures at different levels in the lumbar spine. PET images were reconstructed with OSEM. Boxplots show Q_1_, median, and Q_3_ values.

Figure S4. SUV_max_ normalized against subcutaneous fat (NSUV_max, subcutaneous_) for spinal structures at different levels in the lumbar spine. PET images were reconstructed with OSEM. Boxplots show Q_1_, median, and Q_3_ values.

Figure S5. SUV_mean_ normalized against subcutaneous fat (NSUV_mean, subcutaneous_) for spinal structures at different levels in the lumbar spine. PET images were reconstructed with OSEM. Boxplots show Q_1_, median, and Q_3_ values.
